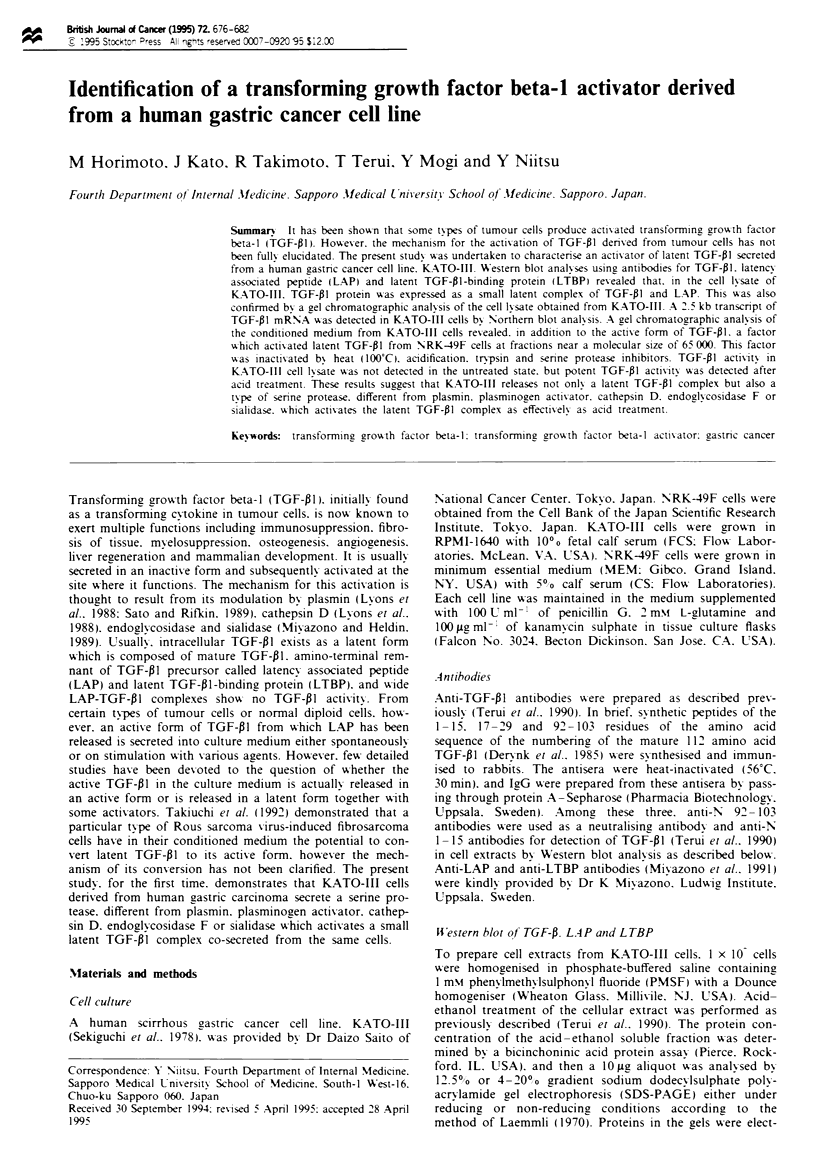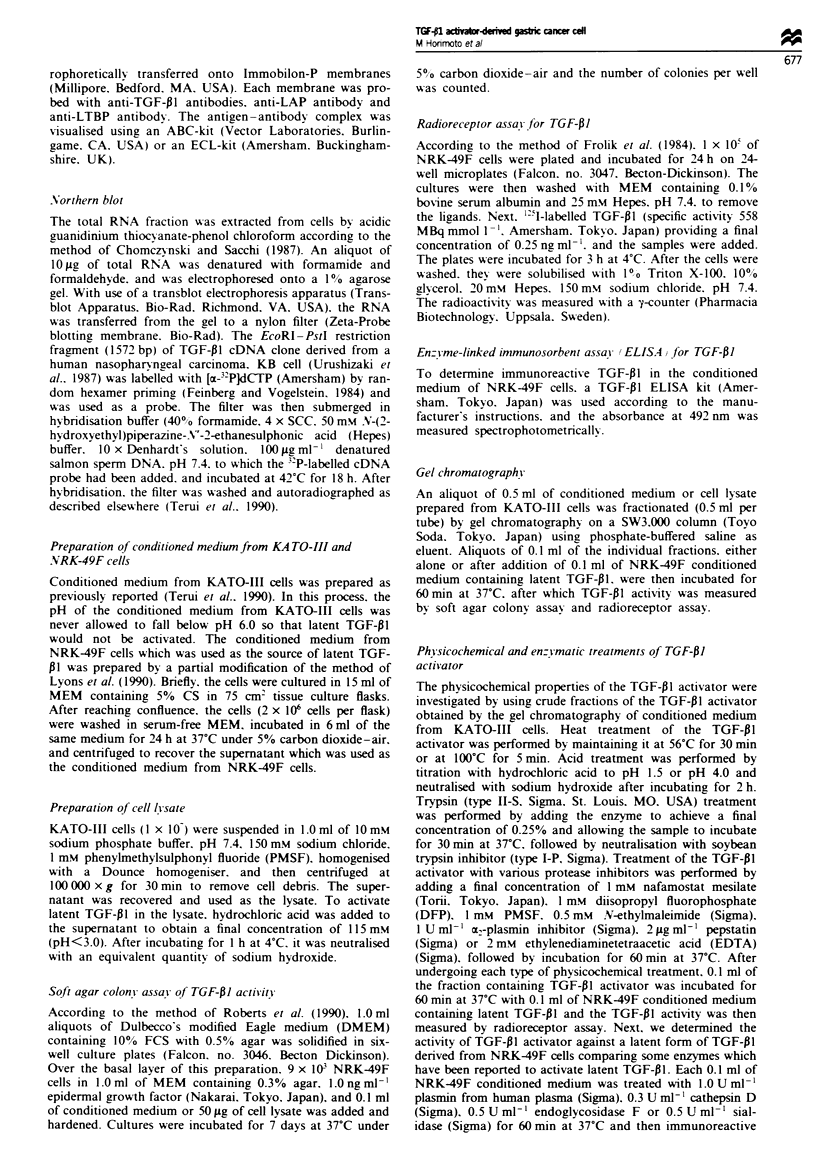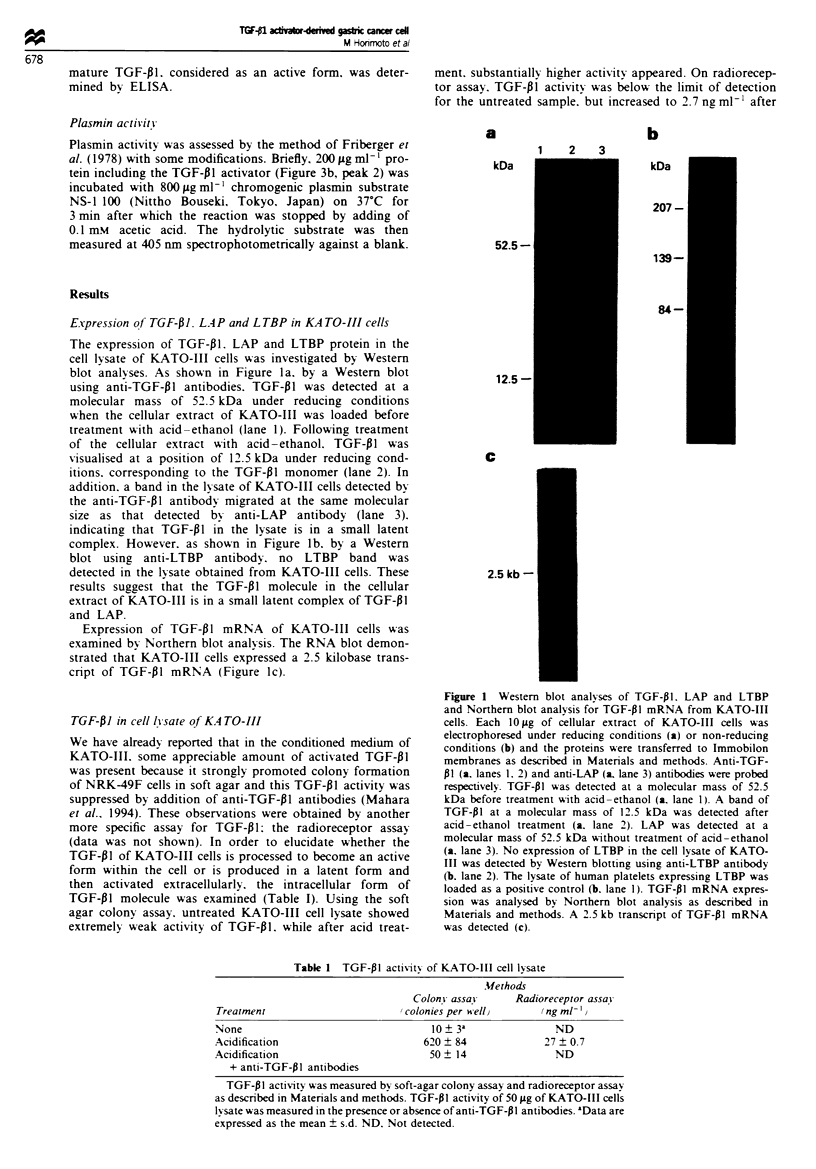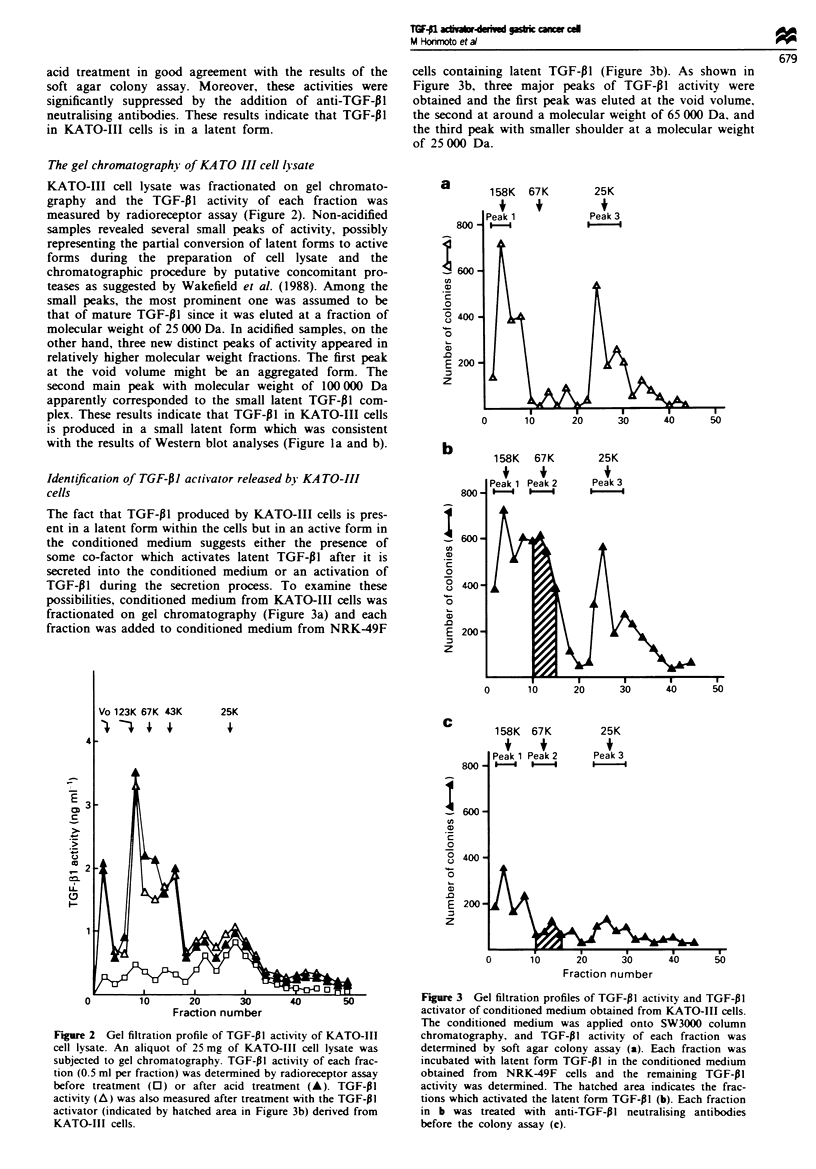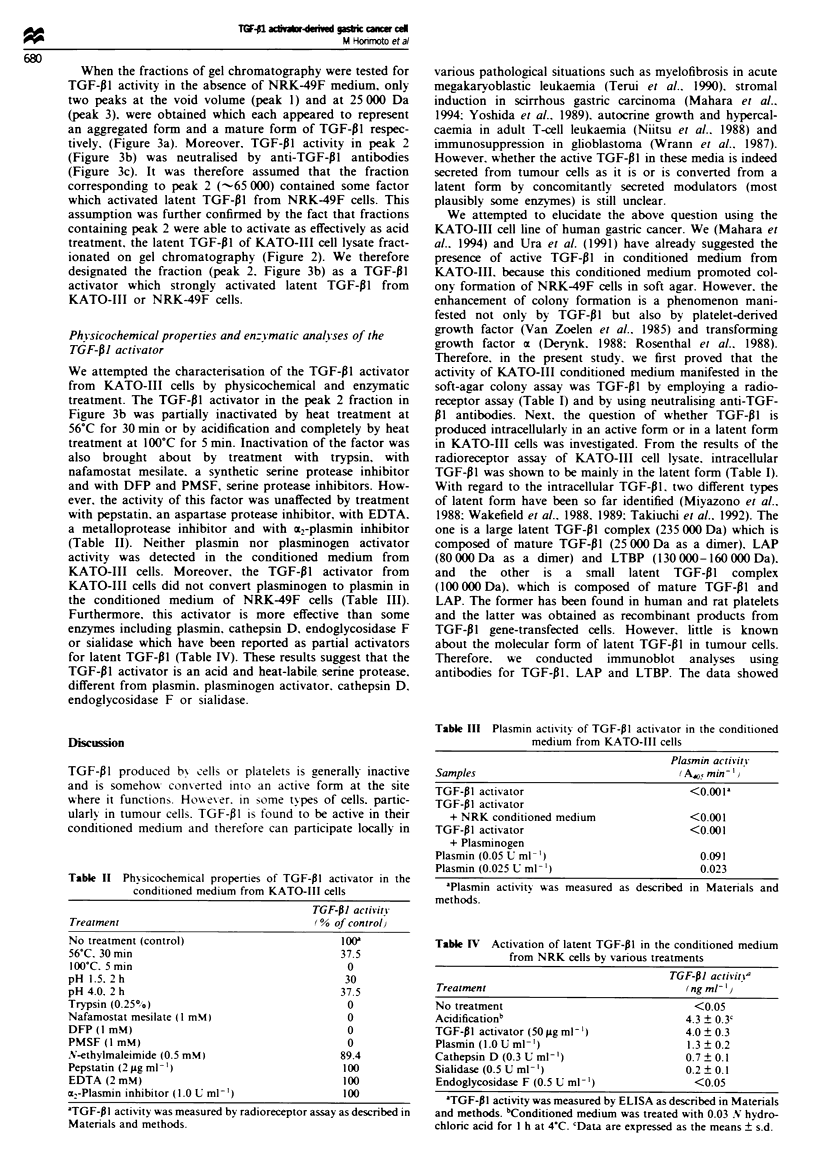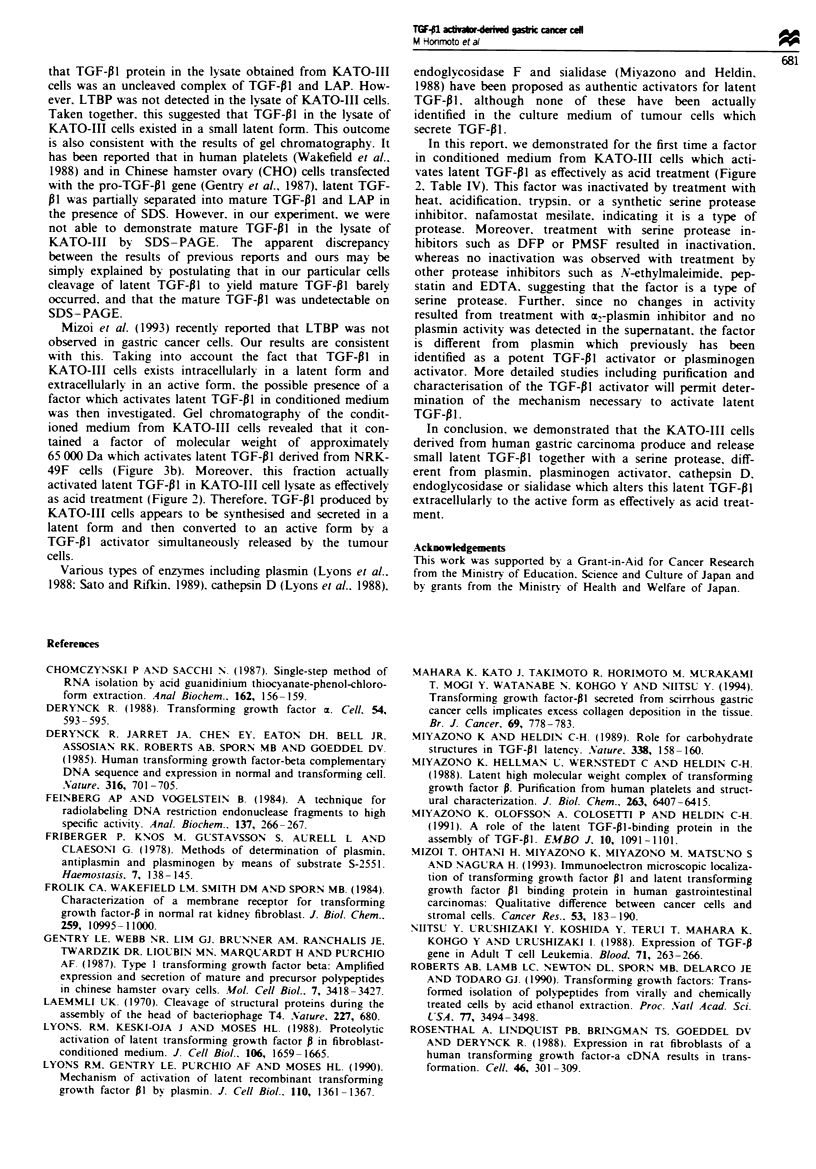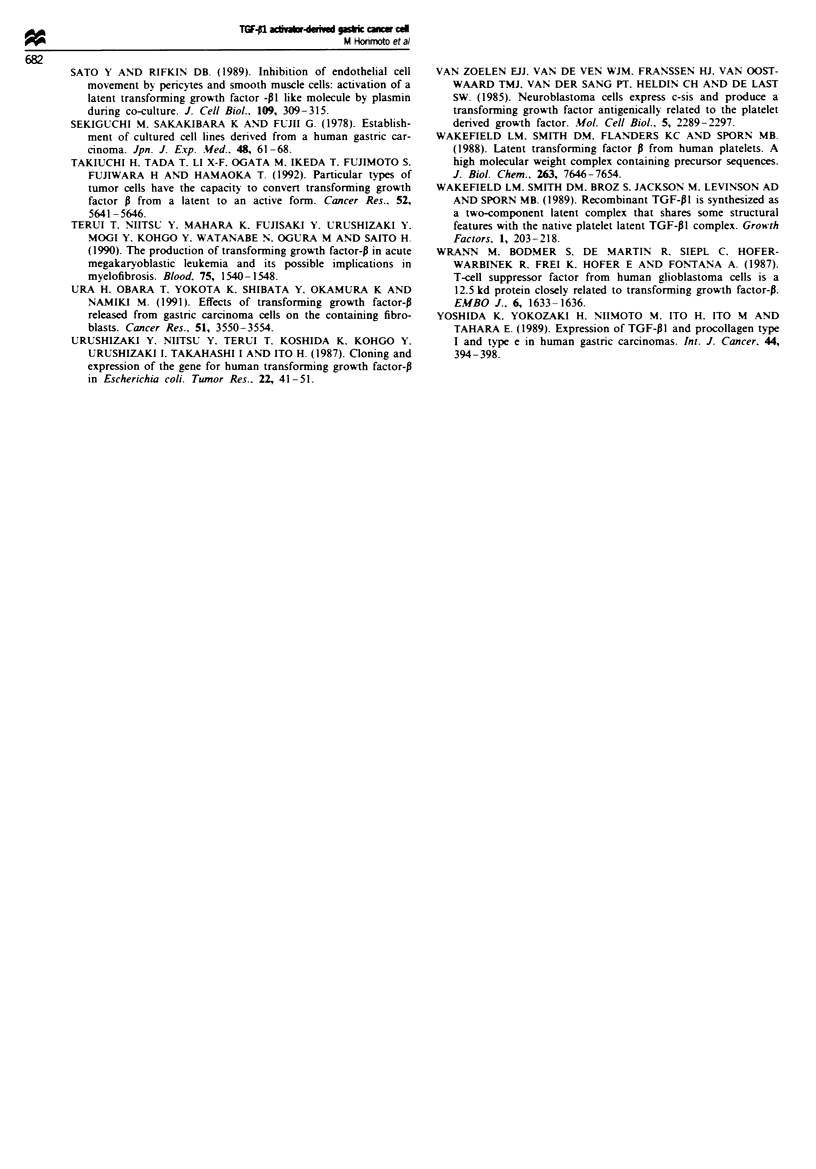# Identification of a transforming growth factor beta-1 activator derived from a human gastric cancer cell line.

**DOI:** 10.1038/bjc.1995.393

**Published:** 1995-09

**Authors:** M. Horimoto, J. Kato, R. Takimoto, T. Terui, Y. Mogi, Y. Niitsu

**Affiliations:** Fourth Department of Internal Medicine, Sapporo Medical University School of Medicine, Japan.

## Abstract

**Images:**


British Journal of Cancer (1995) 72, 676-682

© 1995 Stockton Press All rights reserved 0007-0920/95 $12.00

### Identification of a transforming growth factor beta-1 activator derived from a human gastric cancer cell line

M Horimoto, J Kato, R Takimoto, T Terui, Y Mogi and Y Niitsu

*Fourth Department of Internal Medicine, Sapporo Medical University School of Medicine, Sapporo, Japan.*

**Summary** It has been shown that some types of tumour cells produce activated transforming growth factor beta-1 (TGF-$\beta$1). However, the mechanism for the activation of TGF-$\beta$1 derived from tumour cells has not been fully elucidated. The present study was undertaken to characterise an activator of latent TGF-$\beta$1 secreted from a human gastric cancer cell line, KATO-III. Western blot analyses using antibodies for TGF-$\beta$1, latency associated peptide (LAP) and latent TGF-$\beta$1-binding protein (LTBP) revealed that, in the cell lysate of KATO-III, TGF-$\beta$1 protein was expressed as a small latent complex of TGF-$\beta$1 and LAP. This was also confirmed by a gel chromatographic analysis of the cell lysate obtained from KATO-III. A 2.5 kb transcript of TGF-$\beta$1 mRNA was detected in KATO-III cells by Northern blot analysis. A gel chromatographic analysis of the conditioned medium from KATO-III cells revealed, in addition to the active form of TGF-$\beta$1, a factor which activated latent TGF-$\beta$1 from NRK-49F cells at fractions near a molecular size of 65 000. This factor was inactivated by heat (100°C), acidification, trypsin and serine protease inhibitors. TGF-$\beta$1 activity in KATO-III cell lysate was not detected in the untreated state, but potent TGF-$\beta$1 activity was detected after acid treatment. These results suggest that KATO-III releases not only a latent TGF-$\beta$1 complex but also a type of serine protease, different from plasmin, plasminogen activator, cathepsin D, endoglycosidase F or sialidase, which activates the latent TGF-$\beta$1 complex as effectively as acid treatment.

**Keywords:** transforming growth factor beta-1; transforming growth factor beta-1 activator; gastric cancer

Transforming growth factor beta-1 (TGF-$\beta$1), initially found as a transforming cytokine in tumour cells, is now known to exert multiple functions including immunosuppression, fibrosis of tissue, myelosuppression, osteogenesis, angiogenesis, liver regeneration and mammalian development. It is usually secreted in an inactive form and subsequently activated at the site where it functions. The mechanism for this activation is thought to result from its modulation by plasmin (Lyons *et al.*, 1988; Sato and Rifkin, 1989), cathepsin D (Lyons *et al.*, 1988), endoglycosidase and sialidase (Miyazono and Heldin, 1989). Usually, intracellular TGF-$\beta$1 exists as a latent form which is composed of mature TGF-$\beta$1, amino-terminal remnant of TGF-$\beta$1 precursor called latency associated peptide (LAP) and latent TGF-$\beta$1-binding protein (LTBP), and wide LAP-TGF-$\beta$1 complexes show no TGF-$\beta$1 activity. From certain types of tumour cells or normal diploid cells, however, an active form of TGF-$\beta$1 from which LAP has been released is secreted into culture medium either spontaneously or on stimulation with various agents. However, few detailed studies have been devoted to the question of whether the active TGF-$\beta$1 in the culture medium is actually released in an active form or is released in a latent form together with some activators. Takiuchi *et al.* (1992) demonstrated that a particular type of Rous sarcoma virus-induced fibrosarcoma cells have in their conditioned medium the potential to convert latent TGF-$\beta$1 to its active form, however the mechanism of its conversion has not been clarified. The present study, for the first time, demonstrates that KATO-III cells derived from human gastric carcinoma secrete a serine protease, different from plasmin, plasminogen activator, cathepsin D, endoglycosidase F or sialidase which activates a small latent TGF-$\beta$1 complex co-secreted from the same cells.

### Materials and methods

### *Cell culture*

A human scirrhous gastric cancer cell line, KATO-III (Sekiguchi *et al.*, 1978), was provided by Dr Daizo Saito of National Cancer Center, Tokyo, Japan. NRK-49F cells were obtained from the Cell Bank of the Japan Scientific Research Institute, Tokyo, Japan. KATO-III cells were grown in RPMI-1640 with 10% fetal calf serum (FCS; Flow Laboratories, McLean, VA, USA). NRK-49F cells were grown in minimum essential medium (MEM; Gibco, Grand Island, NY, USA) with 5% calf serum (CS; Flow Laboratories). Each cell line was maintained in the medium supplemented with 100 U $ml^{-1}$ of penicillin G, 2 mM L-glutamine and 100 $\mu$g $ml^{-1}$ of kanamycin sulphate in tissue culture flasks (Falcon No. 3024, Becton Dickinson, San Jose, CA, USA).

### *Antibodies*

Anti-TGF-$\beta$1 antibodies were prepared as described previously (Terui *et al.*, 1990). In brief, synthetic peptides of the 1-15, 17-29 and 92-103 residues of the amino acid sequence of the numbering of the mature 112 amino acid TGF-$\beta$1 (Derynk *et al.*, 1985) were synthesised and immunised to rabbits. The antisera were heat-inactivated (56°C, 30 min), and IgG were prepared from these antisera by passing through protein A-Sepharose (Pharmacia Biotechnology, Uppsala, Sweden). Among these three, anti-N 92-103 antibodies were used as a neutralising antibody and anti-N 1-15 antibodies for detection of TGF-$\beta$1 (Terui *et al.*, 1990) in cell extracts by Western blot analysis as described below. Anti-LAP and anti-LTBP antibodies (Miyazono *et al.*, 1991) were kindly provided by Dr K Miyazono, Ludwig Institute, Uppsala, Sweden.

### *Western blot of TGF-β, LAP and LTBP*

To prepare cell extracts from KATO-III cells, $1 \times 10^7$ cells were homogenised in phosphate-buffered saline containing 1 mM phenylmethylsulphonyl fluoride (PMSF) with a Dounce homogeniser (Wheaton Glass, Millivile, NJ, USA). Acid-ethanol treatment of the cellular extract was performed as previously described (Terui *et al.*, 1990). The protein concentration of the acid-ethanol soluble fraction was determined by a bicinchoninic acid protein assay (Pierce, Rockford, IL, USA), and then a 10 $\mu$g aliquot was analysed by 12.5% or 4-20% gradient sodium dodecylsulphate polyacrylamide gel electrophoresis (SDS-PAGE) either under reducing or non-reducing conditions according to the method of Laemmli (1970). Proteins in the gels were elect-

Correspondence: Y Niitsu, Fourth Department of Internal Medicine, Sapporo Medical University School of Medicine, South-1 West-16, Chuo-ku Sapporo 060, Japan
Received 30 September 1994; revised 5 April 1995; accepted 28 April 1995

rophoretically transferred onto Immobilon-P membranes (Millipore, Bedford, MA, USA). Each membrane was probed with anti-TGF-β1 antibodies, anti-LAP antibody and anti-LTBP antibody. The antigen-antibody complex was visualised using an ABC-kit (Vector Laboratories, Burlingame, CA, USA) or an ECL-kit (Amersham, Buckinghamshire, UK).

*Northern blot*

The total RNA fraction was extracted from cells by acidic guanidinium thiocyanate-phenol chloroform according to the method of Chomczynski and Sacchi (1987). An aliquot of 10 μg of total RNA was denatured with formamide and formaldehyde, and was electrophoresed onto a 1% agarose gel. With use of a transblot electrophoresis apparatus (Trans-blot Apparatus, Bio-Rad, Richmond, VA, USA), the RNA was transferred from the gel to a nylon filter (Zeta-Probe blotting membrane, Bio-Rad). The *Eco*RI–*Pst*I restriction fragment (1572 bp) of TGF-β1 cDNA clone derived from a human nasopharyngeal carcinoma, KB cell (Urushizaki *et al.*, 1987) was labelled with [α-$^{32}$P]dCTP (Amersham) by random hexamer priming (Feinberg and Vogelstein, 1984) and was used as a probe. The filter was then submerged in hybridisation buffer (40% formamide, 4 × SCC, 50 mM *N*-(2-hydroxyethyl)piperazine-*N'*-2-ethanesulphonic acid (Hepes) buffer, 10 × Denhardt's solution, 100 μg ml$^{-1}$ denatured salmon sperm DNA, pH 7.4, to which the $^{32}$P-labelled cDNA probe had been added, and incubated at 42°C for 18 h. After hybridisation, the filter was washed and autoradiographed as described elsewhere (Terui *et al.*, 1990).

*Preparation of conditioned medium from KATO-III and NRK-49F cells*

Conditioned medium from KATO-III cells was prepared as previously reported (Terui *et al.*, 1990). In this process, the pH of the conditioned medium from KATO-III cells was never allowed to fall below pH 6.0 so that latent TGF-β1 would not be activated. The conditioned medium from NRK-49F cells which was used as the source of latent TGF-β1 was prepared by a partial modification of the method of Lyons *et al.* (1990). Briefly, the cells were cultured in 15 ml of MEM containing 5% CS in 75 cm$^2$ tissue culture flasks. After reaching confluence, the cells (2 × 10$^6$ cells per flask) were washed in serum-free MEM, incubated in 6 ml of the same medium for 24 h at 37°C under 5% carbon dioxide-air, and centrifuged to recover the supernatant which was used as the conditioned medium from NRK-49F cells.

*Preparation of cell lysate*

KATO-III cells (1 × 10$^7$) were suspended in 1.0 ml of 10 mM sodium phosphate buffer, pH 7.4, 150 mM sodium chloride, 1 mM phenylmethylsulphonyl fluoride (PMSF), homogenised with a Dounce homogeniser, and then centrifuged at 100 000 × *g* for 30 min to remove cell debris. The supernatant was recovered and used as the lysate. To activate latent TGF-β1 in the lysate, hydrochloric acid was added to the supernatant to obtain a final concentration of 115 mM (pH<3.0). After incubating for 1 h at 4°C, it was neutralised with an equivalent quantity of sodium hydroxide.

*Soft agar colony assay of TGF-β1 activity*

According to the method of Roberts *et al.* (1990), 1.0 ml aliquots of Dulbecco's modified Eagle medium (DMEM) containing 10% FCS with 0.5% agar was solidified in six-well culture plates (Falcon, no. 3046, Becton Dickinson). Over the basal layer of this preparation, 9 × 10$^3$ NRK-49F cells in 1.0 ml of MEM containing 0.3% agar, 1.0 ng ml$^{-1}$ epidermal growth factor (Nakarai, Tokyo, Japan), and 0.1 ml of conditioned medium or 50 μg of cell lysate was added and hardened. Cultures were incubated for 7 days at 37°C under 5% carbon dioxide-air and the number of colonies per well was counted.

*Radioreceptor assay for TGF-β1*

According to the method of Frolik *et al.* (1984), 1 × 10$^5$ of NRK-49F cells were plated and incubated for 24 h on 24-well microplates (Falcon, no. 3047, Becton-Dickinson). The cultures were then washed with MEM containing 0.1% bovine serum albumin and 25 mM Hepes, pH 7.4, to remove the ligands. Next, $^{125}$I-labelled TGF-β1 (specific activity 558 MBq mmol l$^{-1}$, Amersham, Tokyo, Japan) providing a final concentration of 0.25 ng ml$^{-1}$, and the samples were added. The plates were incubated for 3 h at 4°C. After the cells were washed, they were solubilised with 1% Triton X-100, 10% glycerol, 20 mM Hepes, 150 mM sodium chloride, pH 7.4. The radioactivity was measured with a γ-counter (Pharmacia Biotechnology, Uppsala, Sweden).

*Enzyme-linked immunosorbent assay (ELISA) for TGF-β1*

To determine immunoreactive TGF-β1 in the conditioned medium of NRK-49F cells, a TGF-β1 ELISA kit (Amersham, Tokyo, Japan) was used according to the manufacturer's instructions, and the absorbance at 492 nm was measured spectrophotometrically.

*Gel chromatography*

An aliquot of 0.5 ml of conditioned medium or cell lysate prepared from KATO-III cells was fractionated (0.5 ml per tube) by gel chromatography on a SW3,000 column (Toyo Soda, Tokyo, Japan) using phosphate-buffered saline as eluent. Aliquots of 0.1 ml of the individual fractions, either alone or after addition of 0.1 ml of NRK-49F conditioned medium containing latent TGF-β1, were then incubated for 60 min at 37°C, after which TGF-β1 activity was measured by soft agar colony assay and radioreceptor assay.

*Physicochemical and enzymatic treatments of TGF-β1 activator*

The physicochemical properties of the TGF-β1 activator were investigated by using crude fractions of the TGF-β1 activator obtained by the gel chromatography of conditioned medium from KATO-III cells. Heat treatment of the TGF-β1 activator was performed by maintaining it at 56°C for 30 min or at 100°C for 5 min. Acid treatment was performed by titration with hydrochloric acid to pH 1.5 or pH 4.0 and neutralised with sodium hydroxide after incubating for 2 h. Trypsin (type II-S, Sigma, St. Louis, MO, USA) treatment was performed by adding the enzyme to achieve a final concentration of 0.25% and allowing the sample to incubate for 30 min at 37°C, followed by neutralisation with soybean trypsin inhibitor (type I-P, Sigma). Treatment of the TGF-β1 activator with various protease inhibitors was performed by adding a final concentration of 1 mM nafamostat mesilate (Torii, Tokyo, Japan), 1 mM diisopropyl fluorophosphate (DFP), 1 mM PMSF, 0.5 mM *N*-ethylmaleimide (Sigma), 1 U ml$^{-1}$ $\alpha_2$-plasmin inhibitor (Sigma), 2 μg ml$^{-1}$ pepstatin (Sigma) or 2 mM ethylenediaminetetraacetic acid (EDTA) (Sigma), followed by incubation for 60 min at 37°C. After undergoing each type of physicochemical treatment, 0.1 ml of the fraction containing TGF-β1 activator was incubated for 60 min at 37°C with 0.1 ml of NRK-49F conditioned medium containing latent TGF-β1 and the TGF-β1 activity was then measured by radioreceptor assay. Next, we determined the activity of TGF-β1 activator against a latent form of TGF-β1 derived from NRK-49F cells comparing some enzymes which have been reported to activate latent TGF-β1. Each 0.1 ml of NRK-49F conditioned medium was treated with 1.0 U ml$^{-1}$ plasmin from human plasma (Sigma), 0.3 U ml$^{-1}$ cathepsin D (Sigma), 0.5 U ml$^{-1}$ endoglycosidase F or 0.5 U ml$^{-1}$ sialidase (Sigma) for 60 min at 37°C and then immunoreactive mature TGF-β1, considered as an active form, was determined by ELISA.

*Plasmin activity*

Plasmin activity was assessed by the method of Friberger *et al.* (1978) with some modifications. Briefly, 200 μg ml$^{-1}$ protein including the TGF-β1 activator (Figure 3b, peak 2) was incubated with 800 μg ml$^{-1}$ chromogenic plasmin substrate NS-1 100 (Nittho Bouseki, Tokyo, Japan) on 37°C for 3 min after which the reaction was stopped by adding of 0.1 mM acetic acid. The hydrolytic substrate was then measured at 405 nm spectrophotometrically against a blank.

### Results

*Expression of TGF-β1, LAP and LTBP in KATO-III cells*

The expression of TGF-β1, LAP and LTBP protein in the cell lysate of KATO-III cells was investigated by Western blot analyses. As shown in Figure 1a, by a Western blot using anti-TGF-β1 antibodies, TGF-β1 was detected at a molecular mass of 52.5 kDa under reducing conditions when the cellular extract of KATO-III was loaded before treatment with acid–ethanol (lane 1). Following treatment of the cellular extract with acid–ethanol, TGF-β1 was visualised at a position of 12.5 kDa under reducing conditions, corresponding to the TGF-β1 monomer (lane 2). In addition, a band in the lysate of KATO-III cells detected by the anti-TGF-β1 antibody migrated at the same molecular size as that detected by anti-LAP antibody (lane 3), indicating that TGF-β1 in the lysate is in a small latent complex. However, as shown in Figure 1b, by a Western blot using anti-LTBP antibody, no LTBP band was detected in the lysate obtained from KATO-III cells. These results suggest that the TGF-β1 molecule in the cellular extract of KATO-III is in a small latent complex of TGF-β1 and LAP.

Expression of TGF-β1 mRNA of KATO-III cells was examined by Northern blot analysis. The RNA blot demonstrated that KATO-III cells expressed a 2.5 kilobase transcript of TGF-β1 mRNA (Figure 1c).

*TGF-β1 in cell lysate of KATO-III*

We have already reported that in the conditioned medium of KATO-III, some appreciable amount of activated TGF-β1 was present because it strongly promoted colony formation of NRK-49F cells in soft agar and this TGF-β1 activity was suppressed by addition of anti-TGF-β1 antibodies (Mahara *et al.*, 1994). These observations were obtained by another more specific assay for TGF-β1: the radioreceptor assay (data was not shown). In order to elucidate whether the TGF-β1 of KATO-III cells is processed to become an active form within the cell or is produced in a latent form and then activated extracellularly, the intracellular form of TGF-β1 molecule was examined (Table I). Using the soft agar colony assay, untreated KATO-III cell lysate showed extremely weak activity of TGF-β1, while after acid treatment, substantially higher activity appeared. On radioreceptor assay, TGF-β1 activity was below the limit of detection for the untreated sample, but increased to 2.7 ng ml$^{-1}$ after

**Figure 1** Western blot analyses of TGF-β1, LAP and LTBP and Northern blot analysis for TGF-β1 mRNA from KATO-III cells. Each 10 μg of cellular extract of KATO-III cells was electrophoresed under reducing conditions (**a**) or non-reducing conditions (**b**) and the proteins were transferred to Immobilon membranes as described in Materials and methods. Anti-TGF-β1 (**a**, lanes 1, 2) and anti-LAP (**a**, lane 3) antibodies were probed respectively. TGF-β1 was detected at a molecular mass of 52.5 kDa before treatment with acid–ethanol (**a**, lane 1). A band of TGF-β1 at a molecular mass of 12.5 kDa was detected after acid–ethanol treatment (**a**, lane 2). LAP was detected at a molecular mass of 52.5 kDa without treatment of acid–ethanol (**a**, lane 3). No expression of LTBP in the cell lysate of KATO-III was detected by Western blotting using anti-LTBP antibody (**b**, lane 2). The lysate of human platelets expressing LTBP was loaded as a positive control (**b**, lane 1). TGF-β1 mRNA expression was analysed by Northern blot analysis as described in Materials and methods. A 2.5 kb transcript of TGF-β1 mRNA was detected (**c**).

**Table 1** TGF-β1 activity of KATO-III cell lysate

| Treatment | *Methods* Colony assay (colonies per well) | Radioreceptor assay (ng ml$^{-1}$) |
|---|---|---|
| None | 10 ± 3[a] | ND |
| Acidification | 620 ± 84 | 27 ± 0.7 |
| Acidification + anti-TGF-β1 antibodies | 50 ± 14 | ND |

TGF-β1 activity was measured by soft-agar colony assay and radioreceptor assay as described in Materials and methods. TGF-β1 activity of 50 μg of KATO-III cells lysate was measured in the presence or absence of anti-TGF-β1 antibodies. [a]Data are expressed as the mean ± s.d. ND, Not detected.

acid treatment in good agreement with the results of the soft agar colony assay. Moreover, these activities were significantly suppressed by the addition of anti-TGF-β1 neutralising antibodies. These results indicate that TGF-β1 in KATO-III cells is in a latent form.

### *The gel chromatography of KATO III cell lysate*

KATO-III cell lysate was fractionated on gel chromatography and the TGF-β1 activity of each fraction was measured by radioreceptor assay (Figure 2). Non-acidified samples revealed several small peaks of activity, possibly representing the partial conversion of latent forms to active forms during the preparation of cell lysate and the chromatographic procedure by putative concomitant proteases as suggested by Wakefield *et al.* (1988). Among the small peaks, the most prominent one was assumed to be that of mature TGF-β1 since it was eluted at a fraction of molecular weight of 25 000 Da. In acidified samples, on the other hand, three new distinct peaks of activity appeared in relatively higher molecular weight fractions. The first peak at the void volume might be an aggregated form. The second main peak with molecular weight of 100 000 Da apparently corresponded to the small latent TGF-β1 complex. These results indicate that TGF-β1 in KATO-III cells is produced in a small latent form which was consistent with the results of Western blot analyses (Figure 1a and b).

### *Identification of TGF-β1 activator released by KATO-III cells*

The fact that TGF-β1 produced by KATO-III cells is present in a latent form within the cells but in an active form in the conditioned medium suggests either the presence of some co-factor which activates latent TGF-β1 after it is secreted into the conditioned medium or an activation of TGF-β1 during the secretion process. To examine these possibilities, conditioned medium from KATO-III cells was fractionated on gel chromatography (Figure 3a) and each fraction was added to conditioned medium from NRK-49F

**Figure 2** Gel filtration profile of TGF-β1 activity of KATO-III cell lysate. An aliquot of 25 mg of KATO-III cell lysate was subjected to gel chromatography. TGF-β1 activity of each fraction (0.5 ml per fraction) was determined by radioreceptor assay before treatment (□) or after acid treatment (▲). TGF-β1 activity (Δ) was also measured after treatment with the TGF-β1 activator (indicated by hatched area in Figure 3b) derived from KATO-III cells.

cells containing latent TGF-β1 (Figure 3b). As shown in Figure 3b, three major peaks of TGF-β1 activity were obtained and the first peak was eluted at the void volume, the second at around a molecular weight of 65 000 Da, and the third peak with smaller shoulder at a molecular weight of 25 000 Da.

**Figure 3** Gel filtration profiles of TGF-β1 activity and TGF-β1 activator of conditioned medium obtained from KATO-III cells. The conditioned medium was applied onto SW3000 column chromatography, and TGF-β1 activity of each fraction was determined by soft agar colony assay (**a**). Each fraction was incubated with latent form TGF-β1 in the conditioned medium obtained from NRK-49F cells and the remaining TGF-β1 activity was determined. The hatched area indicates the fractions which activated the latent form TGF-β1 (**b**). Each fraction in **b** was treated with anti-TGF-β1 neutralising antibodies before the colony assay (**c**).

When the fractions of gel chromatography were tested for TGF-β1 activity in the absence of NRK-49F medium, only two peaks at the void volume (peak 1) and at 25 000 Da (peak 3), were obtained which each appeared to represent an aggregated form and a mature form of TGF-β1 respectively, (Figure 3a). Moreover, TGF-β1 activity in peak 2 (Figure 3b) was neutralised by anti-TGF-β1 antibodies (Figure 3c). It was therefore assumed that the fraction corresponding to peak 2 (~65 000) contained some factor which activated latent TGF-β1 from NRK-49F cells. This assumption was further confirmed by the fact that fractions containing peak 2 were able to activate as effectively as acid treatment, the latent TGF-β1 of KATO-III cell lysate fractionated on gel chromatography (Figure 2). We therefore designated the fraction (peak 2, Figure 3b) as a TGF-β1 activator which strongly activated latent TGF-β1 from KATO-III or NRK-49F cells.

*Physicochemical properties and enzymatic analyses of the TGF-β1 activator*

We attempted the characterisation of the TGF-β1 activator from KATO-III cells by physicochemical and enzymatic treatment. The TGF-β1 activator in the peak 2 fraction in Figure 3b was partially inactivated by heat treatment at 56°C for 30 min or by acidification and completely by heat treatment at 100°C for 5 min. Inactivation of the factor was also brought about by treatment with trypsin, with nafamostat mesilate, a synthetic serine protease inhibitor and with DFP and PMSF, serine protease inhibitors. However, the activity of this factor was unaffected by treatment with pepstatin, an aspartase protease inhibitor, with EDTA, a metalloprotease inhibitor and with $\alpha_2$-plasmin inhibitor (Table II). Neither plasmin nor plasminogen activator activity was detected in the conditioned medium from KATO-III cells. Moreover, the TGF-β1 activator from KATO-III cells did not convert plasminogen to plasmin in the conditioned medium of NRK-49F cells (Table III). Furthermore, this activator is more effective than some enzymes including plasmin, cathepsin D, endoglycosidase F or sialidase which have been reported as partial activators for latent TGF-β1 (Table IV). These results suggest that the TGF-β1 activator is an acid and heat-labile serine protease, different from plasmin, plasminogen activator, cathepsin D, endoglycosidase F or sialidase.

### Discussion

TGF-β1 produced by cells or platelets is generally inactive and is somehow converted into an active form at the site where it functions. However, in some types of cells, particularly in tumour cells, TGF-β1 is found to be active in their conditioned medium and therefore can participate locally in various pathological situations such as myelofibrosis in acute megakaryoblastic leukaemia (Terui *et al.*, 1990), stromal induction in scirrhous gastric carcinoma (Mahara *et al.*, 1994; Yoshida *et al.*, 1989), autocrine growth and hypercalcaemia in adult T-cell leukaemia (Niitsu *et al.*, 1988) and immunosuppression in glioblastoma (Wrann *et al.*, 1987). However, whether the active TGF-β1 in these media is indeed secreted from tumour cells as it is or is converted from a latent form by concomitantly secreted modulators (most plausibly some enzymes) is still unclear.

We attempted to elucidate the above question using the KATO-III cell line of human gastric cancer. We (Mahara *et al.*, 1994) and Ura *et al.* (1991) have already suggested the presence of active TGF-β1 in conditioned medium from KATO-III, because this conditioned medium promoted colony formation of NRK-49F cells in soft agar. However, the enhancement of colony formation is a phenomenon manifested not only by TGF-β1 but also by platelet-derived growth factor (Van Zoelen *et al.*, 1985) and transforming growth factor α (Derynk, 1988; Rosenthal *et al.*, 1988). Therefore, in the present study, we first proved that the activity of KATO-III conditioned medium manifested in the soft-agar colony assay was TGF-β1 by employing a radioreceptor assay (Table I) and by using neutralising anti-TGF-β1 antibodies. Next, the question of whether TGF-β1 is produced intracellularly in an active form or in a latent form in KATO-III cells was investigated. From the results of the radioreceptor assay of KATO-III cell lysate, intracellular TGF-β1 was shown to be mainly in the latent form (Table I). With regard to the intracellular TGF-β1, two different types of latent form have been so far identified (Miyazono *et al.*, 1988; Wakefield *et al.*, 1988, 1989; Takiuchi *et al.*, 1992). The one is a large latent TGF-β1 complex (235 000 Da) which is composed of mature TGF-β1 (25 000 Da as a dimer), LAP (80 000 Da as a dimer) and LTBP (130 000–160 000 Da), and the other is a small latent TGF-β1 complex (100 000 Da), which is composed of mature TGF-β1 and LAP. The former has been found in human and rat platelets and the latter was obtained as recombinant products from TGF-β1 gene-transfected cells. However, little is known about the molecular form of latent TGF-β1 in tumour cells. Therefore, we conducted immunoblot analyses using antibodies for TGF-β1, LAP and LTBP. The data showed

**Table II** Physicochemical properties of TGF-β1 activator in the conditioned medium from KATO-III cells

| *Treatment* | *TGF-β1 activity (% of control)* |
|---|---|
| No treatment (control) | 100[a] |
| 56°C, 30 min | 37.5 |
| 100°C, 5 min | 0 |
| pH 1.5, 2 h | 30 |
| pH 4.0, 2 h | 37.5 |
| Trypsin (0.25%) | 0 |
| Nafamostat mesilate (1 mM) | 0 |
| DFP (1 mM) | 0 |
| PMSF (1 mM) | 0 |
| *N*-ethylmaleimide (0.5 mM) | 89.4 |
| Pepstatin (2 μg ml$^{-1}$) | 100 |
| EDTA (2 mM) | 100 |
| $\alpha_2$-Plasmin inhibitor (1.0 U ml$^{-1}$) | 100 |

[a]TGF-β1 activity was measured by radioreceptor assay as described in Materials and methods.

**Table III** Plasmin activity of TGF-β1 activator in the conditioned medium from KATO-III cells

| *Samples* | *Plasmin activity ($A_{405}$ min$^{-1}$)* |
|---|---|
| TGF-β1 activator | <0.001[a] |
| TGF-β1 activator + NRK conditioned medium | <0.001 |
| TGF-β1 activator + Plasminogen | <0.001 |
| Plasmin (0.05 U ml$^{-1}$) | 0.091 |
| Plasmin (0.025 U ml$^{-1}$) | 0.023 |

[a]Plasmin activity was measured as described in Materials and methods.

**Table IV** Activation of latent TGF-β1 in the conditioned medium from NRK cells by various treatments

| *Treatment* | *TGF-β1 activity[a] (ng ml$^{-1}$)* |
|---|---|
| No treatment | <0.05 |
| Acidification[b] | 4.3 ± 0.3[c] |
| TGF-β1 activator (50 μg ml$^{-1}$) | 4.0 ± 0.3 |
| Plasmin (1.0 U ml$^{-1}$) | 1.3 ± 0.2 |
| Cathepsin D (0.3 U ml$^{-1}$) | 0.7 ± 0.1 |
| Sialidase (0.5 U ml$^{-1}$) | 0.2 ± 0.1 |
| Endoglycosidase F (0.5 U ml$^{-1}$) | <0.05 |

[a]TGF-β1 activity was measured by ELISA as described in Materials and methods. [b]Conditioned medium was treated with 0.03 N hydrochloric acid for 1 h at 4°C. [c]Data are expressed as the means ± s.d.

that TGF-β1 protein in the lysate obtained from KATO-III cells was an uncleaved complex of TGF-β1 and LAP. However, LTBP was not detected in the lysate of KATO-III cells. Taken together, this suggested that TGF-β1 in the lysate of KATO-III cells existed in a small latent form. This outcome is also consistent with the results of gel chromatography. It has been reported that in human platelets (Wakefield *et al.*, 1988) and in Chinese hamster ovary (CHO) cells transfected with the pro-TGF-β1 gene (Gentry *et al.*, 1987), latent TGF-β1 was partially separated into mature TGF-β1 and LAP in the presence of SDS. However, in our experiment, we were not able to demonstrate mature TGF-β1 in the lysate of KATO-III by SDS-PAGE. The apparent discrepancy between the results of previous reports and ours may be simply explained by postulating that in our particular cells cleavage of latent TGF-β1 to yield mature TGF-β1 barely occurred, and that the mature TGF-β1 was undetectable on SDS-PAGE.

Mizoi *et al.* (1993) recently reported that LTBP was not observed in gastric cancer cells. Our results are consistent with this. Taking into account the fact that TGF-β1 in KATO-III cells exists intracellularly in a latent form and extracellularly in an active form, the possible presence of a factor which activates latent TGF-β1 in conditioned medium was then investigated. Gel chromatography of the conditioned medium from KATO-III cells revealed that it contained a factor of molecular weight of approximately 65 000 Da which activates latent TGF-β1 derived from NRK-49F cells (Figure 3b). Moreover, this fraction actually activated latent TGF-β1 in KATO-III cell lysate as effectively as acid treatment (Figure 2). Therefore, TGF-β1 produced by KATO-III cells appears to be synthesised and secreted in a latent form and then converted to an active form by a TGF-β1 activator simultaneously released by the tumour cells.

Various types of enzymes including plasmin (Lyons *et al.*, 1988; Sato and Rifkin, 1989), cathepsin D (Lyons *et al.*, 1988), endoglycosidase F and sialidase (Miyazono and Heldin, 1988) have been proposed as authentic activators for latent TGF-β1, although none of these have been actually identified in the culture medium of tumour cells which secrete TGF-β1.

In this report, we demonstrated for the first time a factor in conditioned medium from KATO-III cells which activates latent TGF-β1 as effectively as acid treatment (Figure 2, Table IV). This factor was inactivated by treatment with heat, acidification, trypsin, or a synthetic serine protease inhibitor, nafamostat mesilate, indicating it is a type of protease. Moreover, treatment with serine protease inhibitors such as DFP or PMSF resulted in inactivation, whereas no inactivation was observed with treatment by other protease inhibitors such as *N*-ethylmaleimide, pepstatin and EDTA, suggesting that the factor is a type of serine protease. Further, since no changes in activity resulted from treatment with $\alpha_2$-plasmin inhibitor and no plasmin activity was detected in the supernatant, the factor is different from plasmin which previously has been identified as a potent TGF-β1 activator or plasminogen activator. More detailed studies including purification and characterisation of the TGF-β1 activator will permit determination of the mechanism necessary to activate latent TGF-β1.

In conclusion, we demonstrated that the KATO-III cells derived from human gastric carcinoma produce and release small latent TGF-β1 together with a serine protease, different from plasmin, plasminogen activator, cathepsin D, endoglycosidase or sialidase which alters this latent TGF-β1 extracellularly to the active form as effectively as acid treatment.

**Acknowledgements**
This work was supported by a Grant-in-Aid for Cancer Research from the Ministry of Education, Science and Culture of Japan and by grants from the Ministry of Health and Welfare of Japan.

### References

CHOMCZYNSKI P AND SACCHI N. (1987). Single-step method of RNA isolation by acid guanidinium thiocyanate-phenol-chloroform extraction. *Anal Biochem.*, **162**, 156-159.

DERYNCK R. (1988). Transforming growth factor α. *Cell*, **54**, 593-595.

DERYNCK R, JARRET JA, CHEN EY, EATON DH, BELL JR, ASSOSIAN RK, ROBERTS AB, SPORN MB AND GOEDDEL DV. (1985). Human transforming growth factor-beta complementary DNA sequence and expression in normal and transforming cell. *Nature*, **316**, 701-705.

FEINBERG AP AND VOGELSTEIN B. (1984). A technique for radiolabeling DNA restriction endonuclease fragments to high specific activity. *Anal. Biochem.*, **137**, 266-267.

FRIBERGER P, KNOS M, GUSTAVSSON S, AURELL L AND CLAESONI G. (1978). Methods of determination of plasmin, antiplasmin and plasminogen by means of substrate S-2551. *Haemostasis*, **7**, 138-145.

FROLIK CA, WAKEFIELD LM, SMITH DM AND SPORN MB. (1984). Characterization of a membrane receptor for transforming growth factor-β in normal rat kidney fibroblast. *J. Biol. Chem.*, **259**, 10995-11000.

GENTRY LE, WEBB NR, LIM GJ, BRUNNER AM, RANCHALIS JE, TWARDZIK DR, LIOUBIN MN, MARQUARDT H AND PURCHIO AF. (1987). Type 1 transforming growth factor beta: Amplified expression and secretion of mature and precursor polypeptides in chinese hamster ovary cells. *Mol. Cell Biol.*, **7**, 3418-3427.

LAEMMLI UK. (1970). Cleavage of structural proteins during the assembly of the head of bacteriophage T4. *Nature*, **227**, 680.

LYONS, RM, KESKI-OJA J AND MOSES HL. (1988). Proteolytic activation of latent transforming growth factor β in fibroblast-conditioned medium. *J. Cell Biol.*, **106**, 1659-1665.

LYONS RM, GENTRY LE, PURCHIO AF AND MOSES HL. (1990). Mechanism of activation of latent recombinant transforming growth factor β1 by plasmin. *J. Cell Biol.*, **110**, 1361-1367.

MAHARA K, KATO J, TAKIMOTO R, HORIMOTO M, MURAKAMI T, MOGI Y, WATANABE N, KOHGO Y AND NIITSU Y. (1994). Transforming growth factor-β1 secreted from scirrhous gastric cancer cells implicates excess collagen deposition in the tissue. *Br. J. Cancer*, **69**, 778-783.

MIYAZONO K AND HELDIN C-H. (1989). Role for carbohydrate structures in TGF-β1 latency. *Nature*, **338**, 158-160.

MIYAZONO K, HELLMAN U, WERNSTEDT C AND HELDIN C-H. (1988). Latent high molecular weight complex of transforming growth factor β. Purification from human platelets and structural characterization. *J. Biol. Chem.*, **263**, 6407-6415.

MIYAZONO K, OLOFSSON A, COLOSETTI P AND HELDIN C-H. (1991). A role of the latent TGF-β1-binding protein in the assembly of TGF-β1. *EMBO J*, **10**, 1091-1101.

MIZOI T, OHTANI H, MIYAZONO K, MIYAZONO M, MATSUNO S AND NAGURA H. (1993). Immunoelectron microscopic localization of transforming growth factor β1 and latent transforming growth factor β1 binding protein in human gastrointestinal carcinomas: Qualitative difference between cancer cells and stromal cells. *Cancer Res.*, **53**, 183-190.

NIITSU Y, URUSHIZAKI Y, KOSHIDA Y, TERUI T, MAHARA K, KOHGO Y AND URUSHIZAKI I. (1988). Expression of TGF-β gene in Adult T cell Leukemia. *Blood*, **71**, 263-266.

ROBERTS AB, LAMB LC, NEWTON DL, SPORN MB, DELARCO JE AND TODARO GJ. (1990). Transforming growth factors: Transformed isolation of polypeptides from virally and chemically treated cells by acid ethanol extraction. *Proc. Natl Acad. Sci. USA*, **77**, 3494-3498.

ROSENTHAL A, LINDQUIST PB, BRINGMAN TS, GOEDDEL DV AND DERYNCK R. (1988). Expression in rat fibroblasts of a human transforming growth factor-a cDNA results in transformation. *Cell*, **46**, 301-309.

SATO Y AND RIFKIN DB. (1989). Inhibition of endothelial cell movement by pericytes and smooth muscle cells: activation of a latent transforming growth factor -β1 like molecule by plasmin during co-culture. *J. Cell Biol.*, **109**, 309-315.

SEKIGUCHI M, SAKAKIBARA K AND FUJII G. (1978). Establishment of cultured cell lines derived from a human gastric carcinoma. *Jpn. J. Exp. Med.*, **48**, 61-68.

TAKIUCHI H, TADA T, LI X-F, OGATA M, IKEDA T, FUJIMOTO S, FUJIWARA H AND HAMAOKA T. (1992). Particular types of tumor cells have the capacity to convert transforming growth factor β from a latent to an active form. *Cancer Res.*, **52**, 5641-5646.

TERUI T, NIITSU Y, MAHARA K, FUJISAKI Y, URUSHIZAKI Y, MOGI Y, KOHGO Y, WATANABE N, OGURA M AND SAITO H. (1990). The production of transforming growth factor-β in acute megakaryoblastic leukemia and its possible implications in myelofibrosis. *Blood*, **75**, 1540-1548.

URA H, OBARA T, YOKOTA K, SHIBATA Y, OKAMURA K AND NAMIKI M. (1991). Effects of transforming growth factor-β released from gastric carcinoma cells on the containing fibroblasts. *Cancer Res.*, **51**, 3550-3554.

URUSHIZAKI Y, NIITSU Y, TERUI T, KOSHIDA K, KOHGO Y, URUSHIZAKI I, TAKAHASHI I AND ITO H. (1987). Cloning and expression of the gene for human transforming growth factor-β in *Escherichia coli*. *Tumor Res.*, **22**, 41-51.

VAN ZOELEN EJJ, VAN DE VEN WJM, FRANSSEN HJ, VAN OOSTWAARD TMJ, VAN DER SANG PT, HELDIN CH AND DE LAST SW. (1985). Neuroblastoma cells express c-sis and produce a transforming growth factor antigenically related to the platelet derived growth factor. *Mol. Cell Biol.*, **5**, 2289-2297.

WAKEFIELD LM, SMITH DM, FLANDERS KC AND SPORN MB. (1988). Latent transforming factor β from human platelets. A high molecular weight complex containing precursor sequences. *J. Biol. Chem.*, **263**, 7646-7654.

WAKEFIELD LM, SMITH DM, BROZ S, JACKSON M, LEVINSON AD AND SPORN MB. (1989). Recombinant TGF-β1 is synthesized as a two-component latent complex that shares some structural features with the native platelet latent TGF-β1 complex. *Growth Factors*, **1**, 203-218.

WRANN M, BODMER S, DE MARTIN R, SIEPL C, HOFER-WARBINEK R, FREI K, HOFER E AND FONTANA A. (1987). T-cell suppressor factor from human glioblastoma cells is a 12.5 kd protein closely related to transforming growth factor-β. *EMBO J.*, **6**, 1633-1636.

YOSHIDA K, YOKOZAKI H, NIIMOTO M, ITO H, ITO M AND TAHARA E. (1989). Expression of TGF-β1 and procollagen type I and type e in human gastric carcinomas. *Int. J. Cancer*, **44**, 394-398.